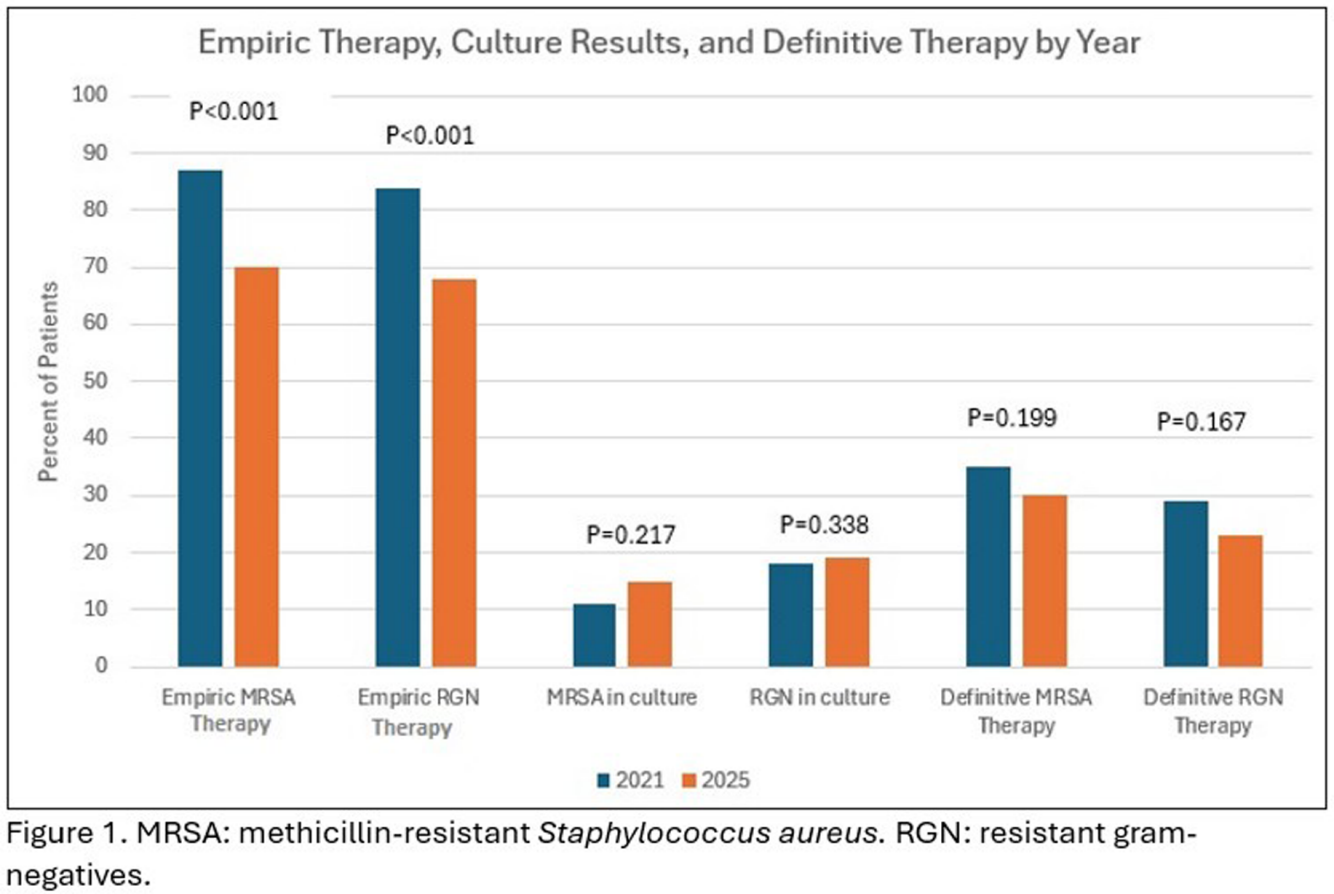# 12 Associations Between Social Determinants of Health and Emergency Department Utilization for Sexually Transmitted Infections

**DOI:** 10.1017/ash.2026.10465

**Published:** 2026-06-23

**Authors:** Morgan Morelli, Andrea Son, Yanis Bitar, Michelle Hecker

**Affiliations:** 1 The MetroHealth System; 2 MetroHealth Medical Center; 3 MetroHealth Hospital

## Abstract

**Background:** Previously published studies, including our own, have demonstrated that empiric antibiotic therapies with activity against methicillin-resistant Staphylococcus aureus (MRSA) and resistant gram-negative (RGN) bacteria are often unnecessarily prescribed for patients hospitalized with diabetic foot infections (DFI) and lower extremity osteomyelitis (OM). We implemented a multidisciplinary intervention based on local and national data to optimize antibiotic prescribing for these patients, including development of a “diabetic foot algorithm,” stakeholder education, and creation of an infectious diseases bone and joint consult service. We evaluated changes in antibiotic use and clinical outcomes after implementation of this intervention. **Methods:** This was a “before and after study” of all patients hospitalized with DFI and/or lower extremity OM in our hospital system in 2021 and 2025. Patients were included if they had an International Classification of Disease, Tenth Revision (ICD-10) diagnosis code of M86, E10.621, E11.621, or E08.621. Patients were excluded if antibiotics were for a different indication or if they were less than 18 years of age. Empiric antibiotic therapy included antibiotics started by the admitting team. Definitive antibiotic therapy included the final antibiotic course either completed during admission or prescribed at the time of discharge. The intervention began in October 2023. The primary outcomes were changes in use of empiric and definitive antibiotic therapies with activity against MRSA and RGN bacteria. The secondary outcomes were changes in amputation rates and hospital length of stay (LOS). **Results:** There were 259 and 242 unique patients hospitalized with DFI or lower extremity OM who met inclusion criteria in 2021 and 2025, respectively. Comparing 2021 to 2025, the percentage of patients receiving empiric therapies with activity against MRSA decreased from 87% to 70% and against RGN bacteria decreased from 84% to 68% (p<0.001) (Figure 1). There was also a trend in the decreased use of definitive therapies with these spectra of activity, while the prevalence of resistant organisms in cultures remained similar. Inpatient days of antibiotic therapy with activity against MRSA and RGN bacteria also decreased 16% and 11%, respectively. There was no difference in clinical outcomes with 44% of patients in both 2021 and 2025 undergoing inpatient amputation surgeries (p=0.997). There was a significant decrease in hospital LOS (8 vs. 6 days, p=0.002). **Conclusions:** A multidisciplinary, multifaceted intervention was associated with significant decreases in use of antibiotic therapies with activity against MRSA and RGN bacteria with no noted adverse effects on clinical outcomes.

**Figure:**